# Genome-Wide Identification and Comprehensive Analysis of *AP2/ERF* Gene Family in *Adiantum nelumboides* Under Abiotic Stress

**DOI:** 10.3390/life15081269

**Published:** 2025-08-11

**Authors:** Di Wu, Tonghua Zhang, Linbao Li, Qianyan Liang, Junchen Wang, Zhiqiang Xiao, Ganju Xiang, Haibo Zhang, Jihong Liu, Guiyun Huang

**Affiliations:** 1 Yangtze River Biodiversity Research Centre, China Three Gorges Corporation, Wuhan 443133, China; wu_di3@ctg.com.cn (D.W.); li_linbao@ctg.com.cn (L.L.); liang_qianyan@ctg.com.cn (Q.L.); wang_junchen@ctg.com.cn (J.W.); xiao_zhiqiang@ctg.com.cn (Z.X.); xiang_ganju@ctg.com.cn (G.X.); zhang_haibo@ctg.com.cn (H.Z.); 2 Hubei Key Laboratory of Rare Resource Plants in Three Gorges Reservoir Area, Yichang 443100, China; 3 National Engineering Research Center of Eco-Environment Protection for Yangtze River Economic Belt, China Three Gorges Corporation, Wuhan 100083, China; 4 National Key Laboratory for Germplasm Innovation & Utilization of Horticultural Crops, College of Horticulture and Forestry Sciences, Huazhong Agricultural University, Wuhan 430070, China; zhangtonghua@mail.hzau.edu.cn (T.Z.); liujihong@mail.hzau.edu.cn (J.L.)

**Keywords:** endangered species, *Adiantum nelumboides*, *AP2/ERF*, abiotic stress, hormone

## Abstract

The *AP2/ERF* (APETALA2/ethylene-responsive element binding factor) family represents one of the largest transcription factor families in plants, playing pivotal roles in abiotic stress responses and hormone signaling pathways. Through genome-wide analysis, we identified 163 *AnAP2/ERF* genes in *Adiantum nelumboides*. Transcriptome data revealed that 12 *AnAP2/ERF* genes were significantly upregulated under either drought or flooding stress, with 8 genes responding to both conditions. qRT-PCR validation confirmed that all 12 selected *AnAP2/ERF* genes exhibited differential expression under both stress types. Notably, these genes also showed significant induction by abscisic acid (ABA), auxin (IAA), and gibberellin (GA), suggesting their potential involvement in stress responses through hormone crosstalk. This study establishes a foundation for investigating *AnAP2/ERF* gene functions and their molecular mechanisms in abiotic stress adaptation in *A. nelumboides*.

## 1. Introduction

Transcription factors (TFs) are sequence-specific DNA-binding proteins that bind to the promoter regions of eukaryotic genes, ensuring the transmission of genetic information from DNA to mRNA in a spatiotemporally specific manner. Typically, transcription factors contain one or more DNA-binding domains, which enable specific DNA recognition and regulation of target gene expression. They play crucial roles in plant growth, development, and responses to environmental stress. To date, approximately 60 transcription factor families have been identified in higher plants, such as *MYB* [1], *WRKY* [2], *NAC* [3] and *AP2/ERF* [4,5].

The *AP2/ERF* transcription factors represent one of the oldest and largest transcription factor families in plant evolution [6,7]. These proteins contain a conserved domain of approximately 60–70 amino acid residues [7,8], and are classified into four subfamilies based on domain number: AP2, ERF, RAV, and Soloist [9,10,11]. Within this classification, ERF and DREB subfamilies mediate plant responses to abiotic stresses including drought, low temperature, and high salinity [12,13]. Transgenic studies demonstrate that overexpression of *AP2/ERF* TFs in rice and *Arabidopsis thaliana* enhances tolerance to both abiotic and biotic stresses [14,15]. *TaERF-6-3A* overexpression lines exhibited enhanced tolerance to drought and salt stress, with concomitant downregulation of stress-responsive and antioxidant genes [16]. Similarly, in rice, *OsAP2/ERF-N22* transgenic lines showed increased relative water content, membrane stability index, cuticular wax deposition, osmotic adjustment capacity, stomatal conductance, and transpiration efficiency [17]. In poplar, *PtoERF15* overexpression maintained stem water potential to confer drought tolerance. Mechanistically, *PtoERF15* and its target *PtoMYC2b* regulate xylem vessel size, density, and cell wall thickness under drought conditions [18].

Moreover, *AP2/ERF* transcription factors integrate hormonal signaling pathways to co-regulate plant growth and abiotic stress responses [19,20,21]. For instance, overexpression of *OsERF71* in rice reduced water loss and enhanced drought tolerance, while simultaneously increasing ABA sensitivity and proline accumulation under exogenous ABA treatment [22]. Overexpression of *OsDREB2B* not only altered plant architecture but also modulated GA biosynthetic gene expression. Similarly, *ZmEREB20* overexpression in *A. thaliana* regulated ABA/GA-related genes, heightened ABA sensitivity, and delayed seed germination under salt stress [23,24]. Overexpression of the *A. thaliana* gene *ERF012* conferred tolerance to temperature, drought, salt, and heavy metal stresses, yet promoted root hair development and accelerated leaf senescence. Notably, exogenous IAA treatment mitigated these phenotypic effects [25]. In *A. thaliana*, *ORA47* overexpression significantly elevated jasmonate (JA) levels, whereas *ERF1* was induced by JA or ethylene. Similarly, *BrERF72* in *Brassica campestris* activated JA biosynthetic genes to regulate leaf senescence [26,27].

*A. nelumboides*, an endemic fern species of Shizhu County (Sichuan Province, China), belongs to the Pteridaceae family [28]. This taxon exhibits distinctive circular to reniform (kidney-shaped) simple leaves and is narrowly distributed in the Yangtze River Basin. Designated as a nationally protected endangered species [29,30], its decline stems from medicinal overharvesting by local communities and habitat destruction due to the Three Gorges Dam construction. As a primitive group of Pteridaceae, *A. nelumboides* has a tetraploid genome and extremely low genetic diversity (H_e_ = 0.232), which provides phytogeographical evidence for the theory of continental drift. Its specific adaptation to acidic and low-phosphorus soils on steep cliffs (with a slope of 65–87°) in the Three Gorges Reservoir Area makes it a key species for maintaining the ecological functions of karst microhabitats [31,32,33]. Its morphological and anatomical features (such as thin cuticle, isobilateral mesophyll, and endodermal structure) further confirm the primitive adaptation strategies to dry and shaded environments [34]. Ecologically adapted to thin-soiled vertical rock faces, *A. nelumboides* endures heterogeneous water availability, ranging from sustained waterlogging to acute drought within microhabitats. In terms of abiotic stress tolerance, this species copes with environmental pressures through multi-level mechanisms: under drought and half-flooding stress, there is an accumulation of metabolites like flavonoids and proline, enhanced activity of antioxidant enzymes, accompanied by differential expression of genes in pathways such as “phenylpropane biosynthesis” and “hormone signal transduction”. Moreover, the reversibility of metabolic and transcriptomic characteristics after rewatering reflects its stress recovery ability [35]. In the early stage, we found that some genes in the *AP2/ERF* family were significantly differentially expressed under drought and flooding stress through transcriptome analysis [35]. However, the functional role of *AP2/ERF* in *A. nelumboides* response to abiotic stress remains unclear. To address this knowledge gap, this study employs bioinformatics approaches to identify the *AP2/ERF* gene family through a genome-wide analysis of *A. nelumboides*. We further characterize the sequence features and potential biological functions of these genes, thereby establishing a theoretical foundation for investigating the role of the *AP2/ERF* gene family in abiotic stress responses. It lays a theoretical foundation for studying the response of *AP2/ERF* gene family to abiotic stress.

## 2. Materials and Methods

### 2.1. Search and Identification of AP2/ERF Gene Sequence in A. nelumboides

Based on the *A. nelumboides* genome (Assembly GCA_022343405.2; NCBI Datasets) [36], we performed BLASTP searches against its proteome using *A. thaliana* AtAP2/ERFs protein sequences (TAIR source) as queries via TBtools (v1.134). Candidate *AP2/ERF* family genes were preliminarily screened, followed by removal of sequences with incomplete domains or redundancy. Conserved domains were validated using NCBI CDD and InterPro, with retention of sequences containing AP2 DNA-binding domains for subsequent analysis. Finally, subcellular localization of these *AP2/ERF* proteins was predicted using ExPASy ProtComp 9.0 and Cell-PLoc 2.0. Physicochemical properties—including coding sequence (CDS) length, molecular weight (Mw), theoretical isoelectric point (pI), and grand average of hydropathicity (GRAVY)—were calculated with ExPASy ProtComp 9.0.

### 2.2. Phylogenetic Analysis of AnAP2/ERF Gene Family

The *AP2/ERF* family protein sequence of *A. thaliana* was downloaded from the TAIR (https://www.arabidopsis.org/) database. The AtAP2/ERFs family protein of *A. thaliana* and the AnAP2/ERFs family protein of *A. nelumboides* were analyzed by MEGA7.0 software, and the obtained phylogenetic tree was further beautified by iTOL (https://itol.embl.de/) online software.

### 2.3. Conserved Domain and Gene Structure Analysis, Cis-Element Analysis of AnAP2/ERF Gene in Adiantum nelumboides

The conserved motifs in *AP2/ERF* protein were predicted by MEME (https://meme-suite.org/meme/tools/meme, accessed on 19 May 2025) online analysis program. The number was set to 10, and other parameters were set to default values. The obtained prediction results were combined with the evolutionary tree of *AnAP2/ERF* family genes of *A. nelumboides*, and visualized using TBtools (v1.134) software. On this basis, the chromosome annotation files of *A. nelumboides* were input to display the structure of *AnAP2/ERF* family genes. TBtools (v1.134) software was used to extract the 2 kb upstream sequence of the *AnAP2/ERFs* gene initiation codon ATG as the promoter region from the genomic data of *A. nelumboides*. The extracted *AnAP2/ERFs* family promoter sequence was uploaded to the Plant Care website (https://bioinformatics.psb.ugent.be/webtools/plantcare/html/, accessed on 19 May 2025) for analysis to obtain cis-acting elements. The *c*is-acting elements analyzed by the software were further analyzed to remove blanks and retain non-biological stress-related elements.

### 2.4. Differential Expression Analysis of AP2/ERF Gene Family in A. nelumboides

Using the *A. nelumboides* transcriptome data from NCBI SRA (BioProject: PRJNA898650) [35], we analyzed *AnAP2/ERFs* family gene expression under drought and flooding stress. Gene expression levels (FPKM) were log_2_-transformed after pseudo-count addition (FPKM + 1) and visualized as heatmaps via TBtools (v1.134).

### 2.5. Plant Material and Treatments

Sporophytes of *A. nelumboides* were sown in peat-based substrate and cultivated in a growth chamber under a 12 h light (140 lx; ~20 μmol·m^−2^·s^−1^)/12 h dark photoperiod at 70% relative humidity and 25 °C. Subsequent experiments were initiated when frond diameter reached 1–2 cm.

(1) Drought Stress Treatment in *A. nelumboides*: Uniform seedlings with optimal growth were selected and divided into experimental and control groups. The control group received regular watering, while the experimental group was irrigated with 250 mL of 20% PEG6000 solution. Both groups were maintained under controlled conditions: 12 h light (140 lx; ~20 μmol·m^−2^·s^−1^)/12 h dark photoperiod, 70% relative humidity, and 25 °C for 48 h. Leaf samples were collected at 0, 6, 12, 24, and 48 h for total RNA extraction [37].
(2) Flooding Stress Treatment in *A. nelumboides*: Uniform seedlings were divided into experimental and control groups. The control group received regular watering, whereas the experimental group was fully submerged in water. Both groups were incubated under identical conditions: 12 h light (140 lx)/12 h dark, 70% humidity, 25 °C for 48 h. Leaf samples were harvested at 0, 6, 12, 24, and 48 h for total RNA isolation [35].
(3) Phytohormone Treatment in *A. nelumboides*: Uniform seedlings were assigned to experimental and control groups. The control group was watered normally, while experimental groups were foliar-sprayed with 100 nM IAA; 100 μM gibberellic acid (GA_3_); 100 μM ABA. All groups were kept under 12 h light (140 lx)/12 h dark, 70% humidity, 25 °C for 48 h. Leaf sampling occurred at 0, 6, 12, 24, and 48 h for total RNA extraction [38].

### 2.6. Total RNA Extraction and Quantitative PCR Analysis

Total RNA was extracted using Vazyme (code: RC411-01) Fast Pure Universal Plant Total RNA Isolation Kit. RNA was used to synthesize cDNA using a HiScript II Q RT SuperMix for qPCR (+gDNA wiper) Reagent Kit from Vazyme (code: R223-01). RT-qPCR was performed on a Rocgene Archimed X4 instrument with ChamQ Universal SYBR QPCR Master Mix reagent (Vazyme: Q711-02) according to manufacturer’s instructions. The fern *40S* was used as the reference gene in this experiment. Each analysis included 3 biological replicates and 3 technical replicates. RT-qPCR primers are shown in Supplementary Table S2.

### 2.7. Data Analysis

Differentially expressed genes (DEGs) were identified using DESeq2 with thresholds of false discovery rate (FDR) < 0.05 and |log2 (fold change)| > 1. Differentially expressed proteins (DEPs) were detected by Student’s *t*-test with |fold change| > 2 and *p*-value < 0.05. Three biological replicates were analyzed per sample. Protein–gene expression correlations were assessed via Pearson’s correlation coefficient. The relative expression of genes was analyzed using the 2^−ΔΔCt^ method, and statistical analysis was performed in GraphPad prism 8.0.2 software, differences in gene expression were detected by Student’s *t* test.

## 3. Results

### 3.1. Identification of AnAP2/ERF Gene Family, Phylogenetic Analysis

**Figure 1:**
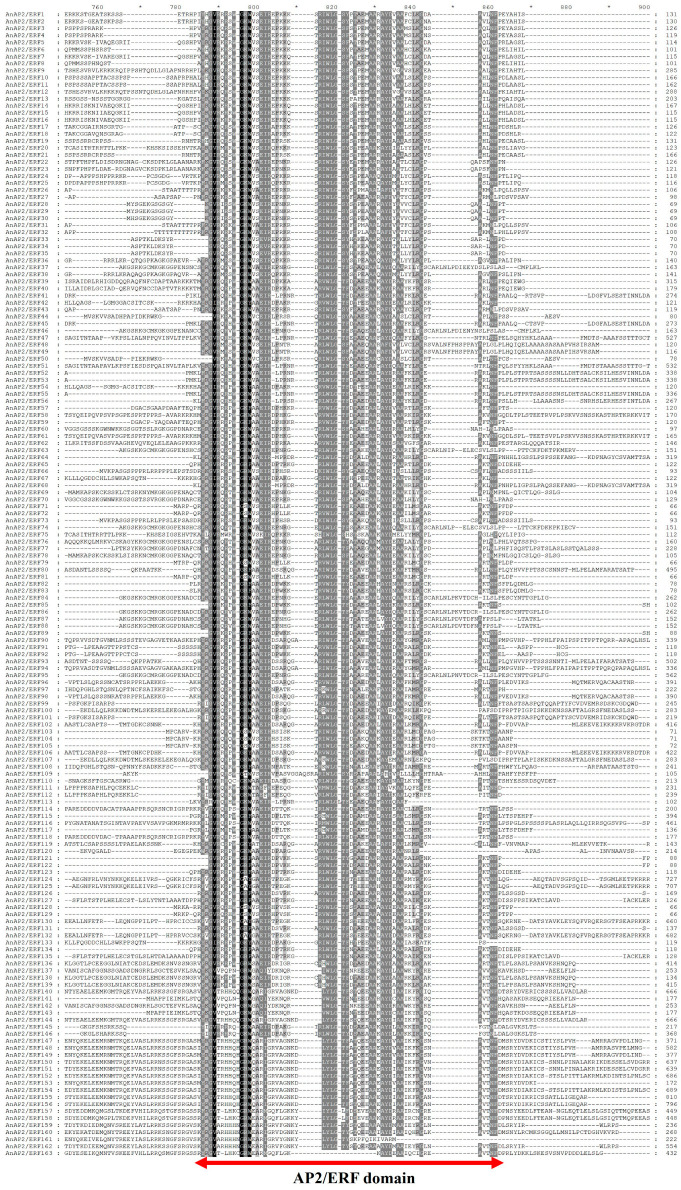
A total of 163 *AnAP2/ERF* family genes were identified from *A. nelumboides*, and their protein sequences were subjected to multiple sequence alignment. The red arrow is marked as *AP2/ERF* domain.

The *A. thaliana AtAP2/ERF* family genes were aligned using TBtools against the genome of *A. nelumboides*. The aligned sequences were screened using NCBI Batch CD-Search, and a total of 163 *AnAP2/ERF* genes were identified, each containing a typical AP2 domain (Figure 1).

**Figure 2:**
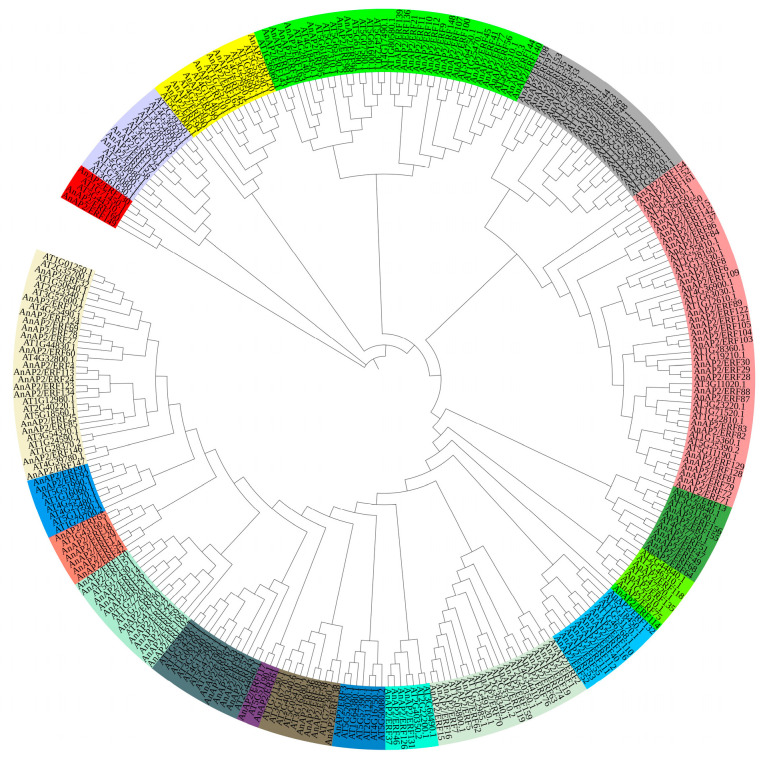
Phylogenetic tree of *AP2/ERF* family genes in *A. nelumboides* and *A. thaliana*.

The physical properties of the proteins encoded by the *AnAP2/ERF* superfamily genes were analyzed, including isoelectric point (pI), molecular weight (MW), CDS length, and subcellular localization (Table S1). The MWs of the predicted proteins varied from 10.8 kDa (AnAP2/ERF35) to 149.9 kDa (AnAP2/ERF124), and the pI values ranged from 4.65 (AnAP2/ERF1, AnAP2/ERF2, AnAP2/ERF101) to 11.15 (AnAP2/ERF113). The outcomes of predicted subcellular localization revealed that all 163 *AnAP2/ERF* members were localized in the nucleus (Table S1), which indicates that they likely play a role in transcriptional regulation. Most of the *AnAP2/ERF* proteins are acidic (pI < 7.0), while 68 are alkaline (pI > 7.0). The predicted average GRAVY score for all AnAP2/ERFs proteins was negative, indicating that they are hydrophilic (Table S1). To examine the evolution of the *AnAP2/ERF* genes, a phylogenetic tree was constructed using the amino acid sequences of the 163 *AnAP2/ERF* proteins and *A. thaliana* At*AP2/ERF* proteins (Figure 2).

### 3.2. Conserved Domain and Gene Structure Analysis of AnAP2/ERFs

**Figure 3:**
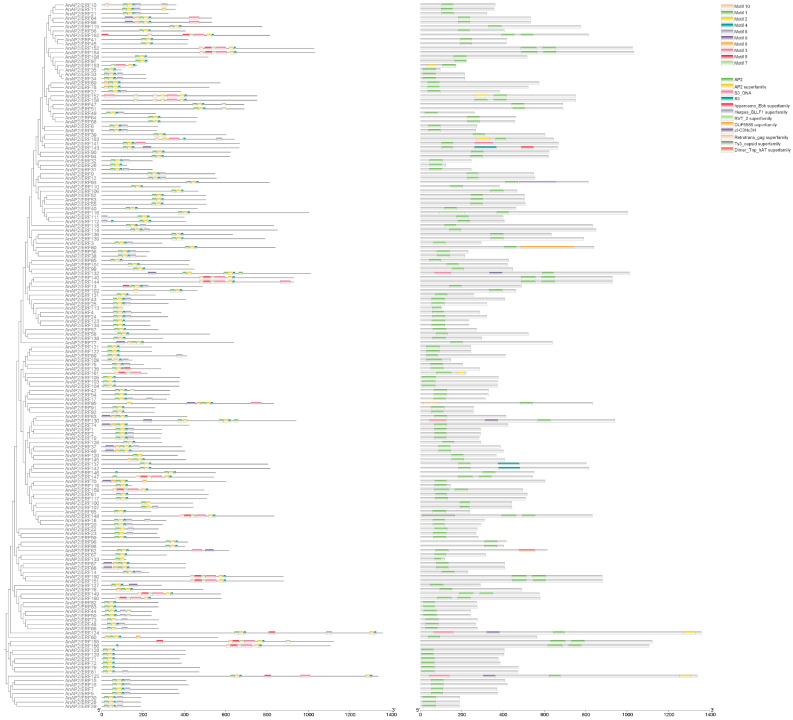
Analysis of evolution, conserved motifs and conserved domains of *AP2/ERF* gene family in *A. nelumboides*.

Gene structure analysis revealed variation in intron number among *AnAP2/ERF* family members. The number of introns ranged from 2 to 11, and the number of exons ranged from 1 to 10. Additionally, some genes lack UTRs (Figure S1). To further study the function of *AnAP2/ERF* family proteins, conserved motifs were analyzed using the MEME suite. Ten motifs were identified, with Motifs 1 and 2 present in all family members. Analysis by NCBI Batch CD-Search confirmed that the AP2 domain is present in all *AnAP2/ERF* family proteins and revealed that most share similar conserved domains in the N-terminal region (Figure 3). Taken together, the results of gene structure, conserved motif, and domain analyses indicate that the *AnAP2/ERFs* gene family is evolutionarily conserved.

### 3.3. Analysis of Cis-Acting Elements of AnAP2/ERFs Family Gene Promoter

**Figure 4:**
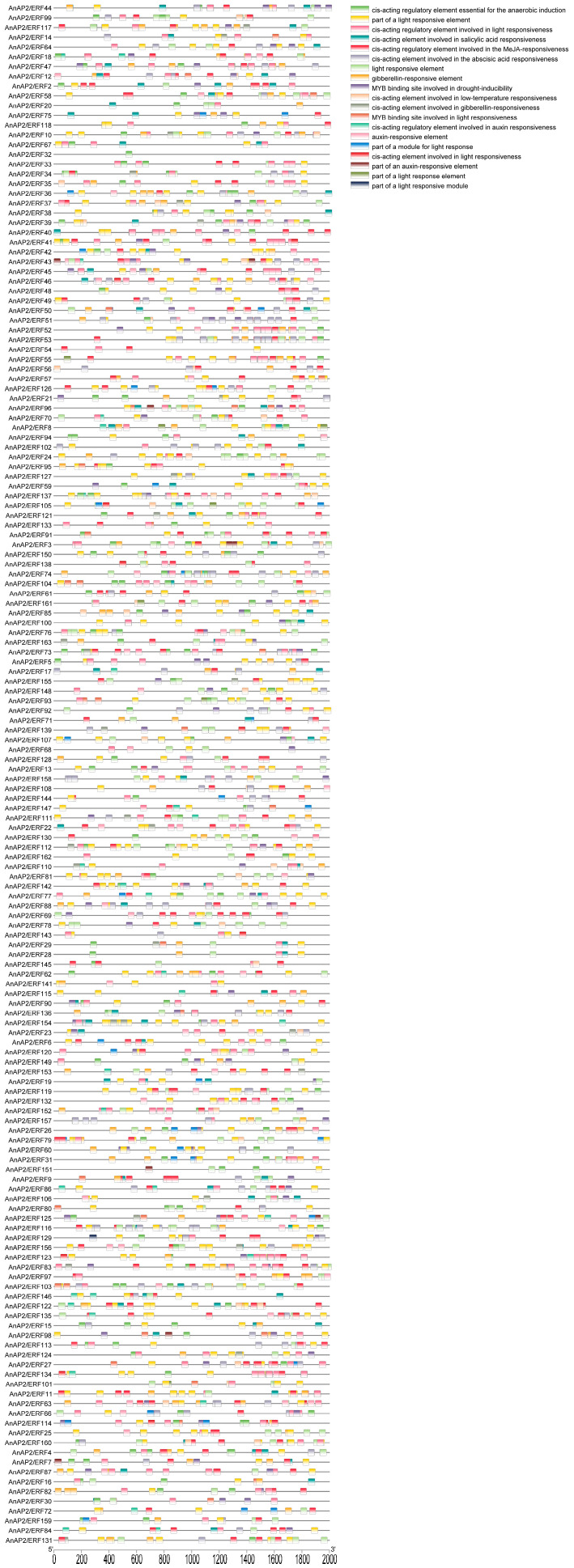
Analysis of promoter *cis*-acting elements of *AP2/ERF* gene family in *A. nelumboides*.

In order to further understand the regulatory roles of the *AnAP2/ERFs* gene family in the growth, development, and stress response of *A. nelumboides*, we analyzed *c*is-acting elements in the 2-kb promoter regions upstream of the transcription start sites of all 163 *AnAP2/ERF* genes. Nineteen *c*is-acting elements associated with hormone responses and abiotic stress were identified. Hormone-response elements included ABRE, TCA-element/SARE, TATC-box/P-box/GARE-motif, TGACG-motif/CGTCA-motif, and TGA-element/AuxRR-core. Stress-related elements included drought, anaerobic, and low-temperature-responsive elements, along with light-responsive elements (Figure 4). These results suggest that *AnAP2/ERF* genes may be regulated by GA, ABA, IAA, and MeJA signaling pathways during development, and could play roles in environmental stress adaptation in *A. nelumboides*.

### 3.4. The Expression Profile of AnAP2/ERF Gene Under Drought and Flooding Stress

**Figure 5:**
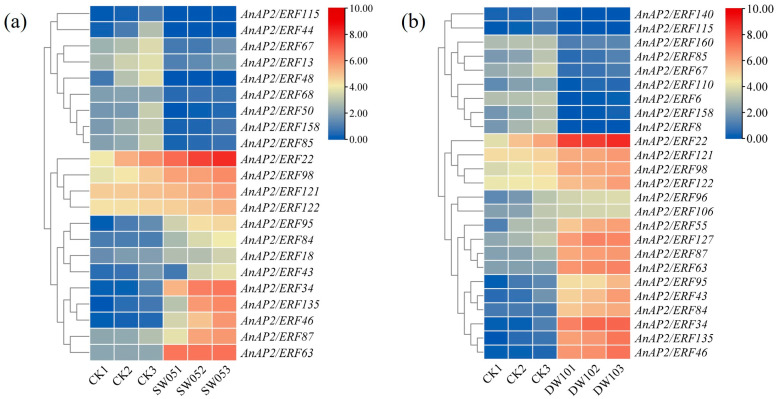
Expression patterns of *AnAP2/ERF* genes in *A. nelumboides* under abiotic stress. (**a**) Differential expression under drought stress for 5 days compared to control (CK). (**b**) Differential expression under flooding stress for 10 days compared to control (CK). Expression values were normalized as log_2_(FPKM + 1) for visualization and represented by a heatmap. Red and blue indicate upregulation and downregulation, respectively, relative to CK. Each cell in the heatmap represents the mean value of three biological replicates. CK: control; SW05: drought for 5 days; DW10: half-flooding for 10 days.

To investigate the function of *AnAP2/ERF* genes in response to drought and flooding stress in *A. nelumboides*, we analyzed their expression patterns using RNA-seq data. Under drought stress, 22 *AnAP2/ERFs* were differentially expressed, with *AnAP2/ERF18*, *AnAP2/ERF22*, *AnAP2/ERF34*, *AnAP2/ERF43*, *AnAP2/ERF46*, *AnAP2/ERF63*, *AnAP2/ERF84*, *AnAP2/ERF87*, *AnAP2/ERF95*, and *AnAP2/ERF135* showing significant upregulation (Figure 5a). Under flooding stress, 25 *AnAP2/ERFs* were differentially expressed, and *AnAP2/ERF22*, *AnAP2/ERF34*, *AnAP2/ERF43*, *AnAP2/ERF46*, *AnAP2/ERF55*, *AnAP2/ERF63*, *AnAP2/ERF84*, *AnAP2/ERF87*, *AnAP2/ERF95*, *AnAP2/ERF96*, *AnAP2/ERF106*, *AnAP2/ERF127*, and *AnAP2/ERF135* were significantly upregulated (Figure 5b).

Notably, *AnAP2/ERF22*, *AnAP2/ERF34*, *AnAP2/ERF43*, *AnAP2/ERF46*, *AnAP2/ERF63*, *AnAP2/ERF84*, *AnAP2/ERF87*, *AnAP2/ERF95*, and *AnAP2/ERF135* were upregulated under both drought and flooding stress (Figure 5). In contrast, *AnAP2/ERF55* and *AnAP2/ERF127* were specifically upregulated under flooding stress, while *AnAP2/ERF18* was specifically upregulated under drought stress (Figure 5). These results suggest that these genes may play potential roles in drought and flooding stress tolerance in *A. nelumboides*.

**Figure 6:**
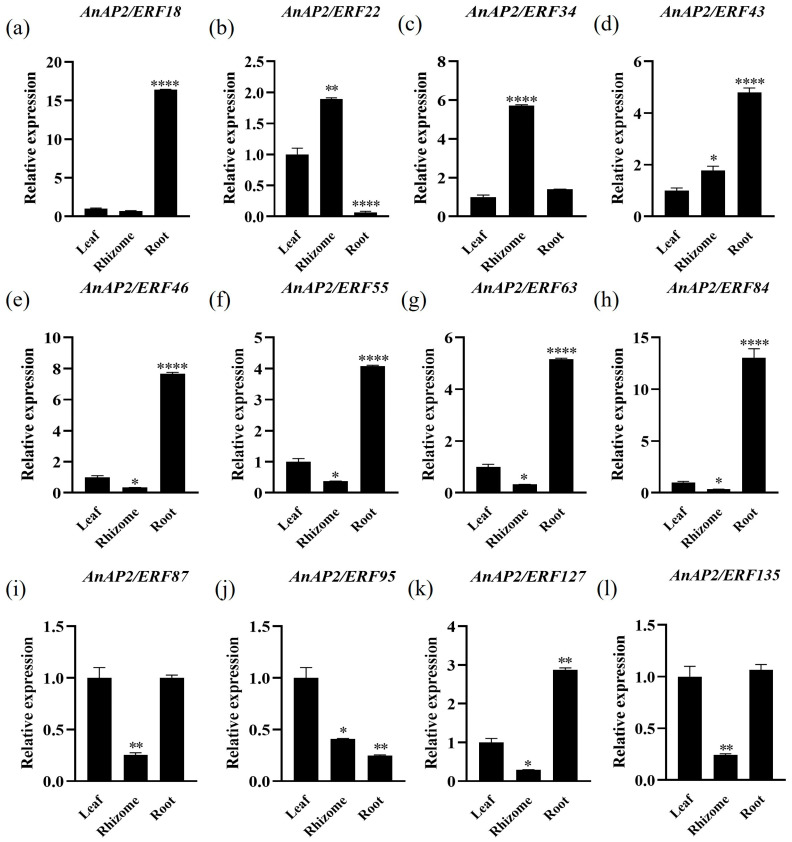
Differential tissue expression of twelve *AnAP2/ERF* genes in *A. nelumboides*. (**a**–**l**) represent the difference of *AnAP2/ERF18*, *AnAP2/ERF22*, *AnAP2/ERF34*, *AnAP2/ERF43*, *AnAP2/ERF46*, *AnAP2/ERF55*, *AnAP2/ERF63*, *AnAP2/ERF84*, *AnAP2/ERF87*, *AnAP2/ERF95*, *AnAP2/ERF127* and *AnAP2/ERF135* in different tissues. Error bars represent SD (3 biological replicates), (*t*-test, * *p* < 0.05, ** *p* < 0.01, **** *p* < 0.0001).

To investigate the roles of 12 *AnAP2/ERF* genes (*AnAP2/ERF18*, *AnAP2/ERF22*, *AnAP2/ERF34*, *AnAP2/ERF43*, *AnAP2/ERF46*, *AnAP2/ERF55*, *AnAP2/ERF63*, *AnAP2/ERF84*, *AnAP2/ERF87*, *AnAP2/ERF95*, *AnAP2/ERF127*, and *AnAP2/ERF135*) in the growth and development of *A. nelumboides*, we analyzed their tissue-specific expression profiles using qRT-PCR. The results demonstrated that all examined *AnAP2/ERFs* genes were expressed across various tissues, suggesting their potential involvement in fundamental growth processes (Figure 6). Distinct tissue-specific patterns were observed: *AnAP2/ERF18*, *AnAP2/ERF43*, *AnAP2/ERF46*, *AnAP2/ERF55*, *AnAP2/ERF63*, *AnAP2/ERF84*, and *AnAP2/ERF127* showed highest expression in roots (Figure 6a,d–h,k); *AnAP2/ERF22* and *AnAP2/ERF34* were predominantly expressed in rhizomes (Figure 6b,c); *AnAP2/ERF87* and *AnAP2/ERF135* exhibited low expression in rhizomes but higher levels in leaves and roots (Figure 6i,l); *AnAP2/ERF95* expression was specifically elevated in leaves and reduced in rhizomes and roots (Figure 6j). These differential expression patterns provide insights for future functional studies on their roles in drought and flooding tolerance.

### 3.5. Expression Analysis of Selected AnAP2/ERFs After Drought and Flooding Stress

**Figure 7:**
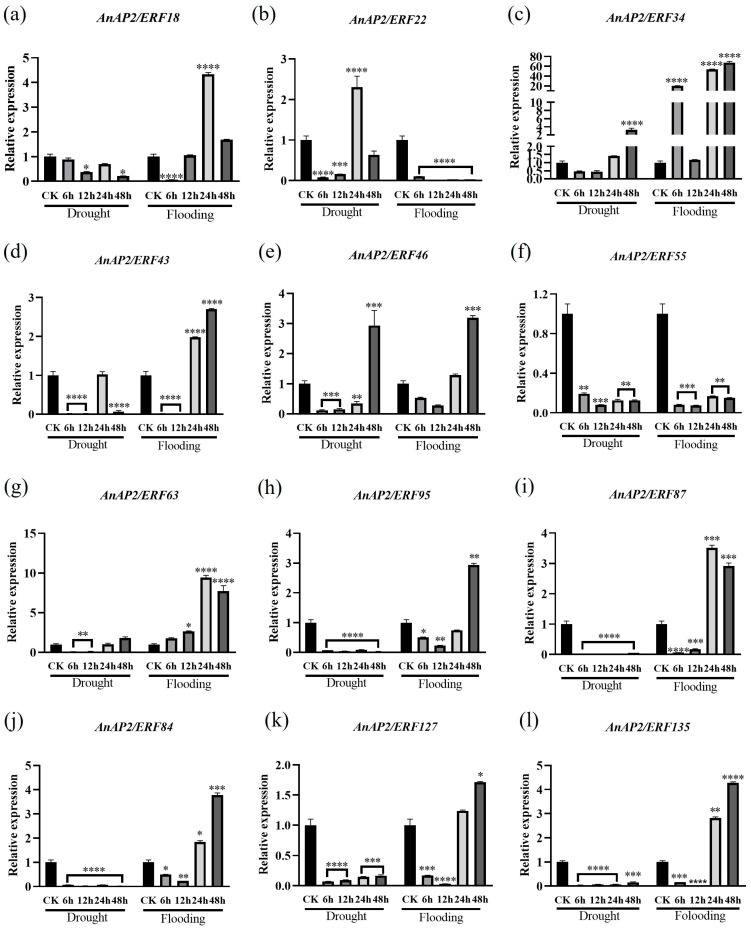
Expression analysis of 12 *AnAP2/ERF* genes induced by drought and flooding stress in *A. nelumboides*. (**a**–**l**) indicated the difference of *AnAP2/ERF18*, *AnAP2/ERF22*, *AnAP2/ERF34*, *AnAP2/ERF43*, *AnAP2/ERF46*, *AnAP2/ERF55*, *AnAP2/ERF63*, *AnAP2/ERF84*, *AnAP2/ERF87*, *AnAP2/ERF95*, *AnAP2/ERF127* and *AnAP2/ERF135* under drought and flooding stress, respectively. Error bars indicate SD (3 biological replicates), (*t*-test, * *p* < 0.05, ** *p* < 0.01, *** *p* < 0.001, **** *p* < 0.0001). The comparisons were in comparison to CK unless otherwise shown in differently in the figure.

To further validate the expression of selected *AnAP2/ERF* genes under drought and flooding stress, healthy *A. nelumboides* plants were subjected to stress treatments with tissue sampling at 0, 6, 12, 24, and 48 h for qRT-PCR analysis. Under drought stress, *AnAP2/ERF18*, *AnAP2/ERF43*, *AnAP2/ERF55*, *AnAP2/ERF84*, *AnAP2/ERF87*, *AnAP2/ERF95*, *AnAP2/ERF127*, and *AnAP2/ERF135* were downregulated relative to CK, while other genes showed upregulation to varying degrees (Figure 7a,d,f,h–l). Under flooding stress, *AnAP2/ERF18*, *AnAP2/ERF34*, *AnAP2/ERF43*, *AnAP2/ERF46*, *AnAP2/ERF63*, *AnAP2/ERF84*, *AnAP2/ERF87*, *AnAP2/ERF95*, *AnAP2/ERF127*, and *AnAP2/ERF135* were upregulated (Figure 7a,c–e,g–l), whereas only *AnAP2/ERF22* and *AnAP2/ERF55* were downregulated (Figure 7b,f). Although qRT-PCR results partially differed from transcriptome data—likely due to variations in experimental timing and biological replicates—both methods confirmed differential expression of these 12 *AnAP2/ERF* genes in response to drought and flooding stress, suggesting their functional roles in stress adaptation.

### 3.6. Expression Analysis of Screened Genes in Response to Hormones

**Figure 8:**
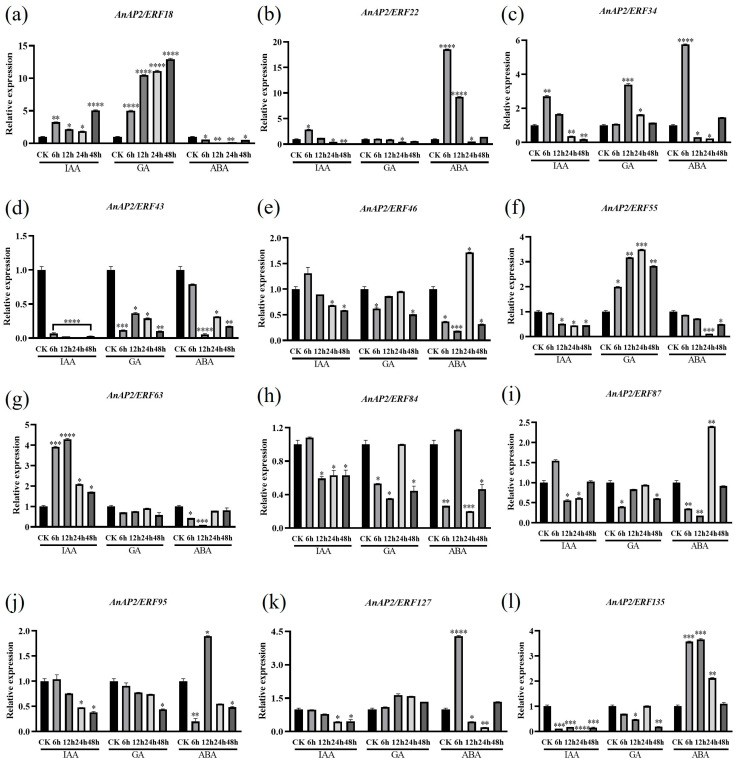
Expression analysis of 12 *AnAP2/ERF* genes induced by IAA, GA and ABA in *A. nelumboides*. (**a**–**l**) indicated the difference of *AnAP2/ERF18*, *AnAP2/ERF22*, *AnAP2/ERF34*, *AnAP2/ERF43*, *AnAP2/ERF46*, *AnAP2/ERF55*, *AnAP2/ERF63*, *AnAP2/ERF84*, *AnAP2/ERF87*, *AnAP2/ERF95*, *AnAP2/ERF127* and *AnAP2/ERF135* under IAA, GA and ABA treatments, respectively. Error bars indicate SD (3 biological replicates), (*t*-test, * *p* < 0.05, ** *p* < 0.01, *** *p* < 0.001, **** *p* < 0.0001). The comparisons were in comparison to CK unless otherwise shown in differently in the figure.

To analyze the response of *AnAP2/ERF* genes to hormones, *A. nelumboides* plants were treated with IAA, GA, and ABA, with samples collected at 0, 6, 12, 24, and 48 h for qRT-PCR expression analysis. The results showed that the 12 selected genes exhibited differential expression under hormone treatments. Under IAA treatment, *AnAP2/ERF18*, *AnAP2/ERF22*, *AnAP2/ERF34*, *AnAP2/ERF46*, *AnAP2/ERF63*, *AnAP2/ERF87* were upregulated relative to CK whereas *AnAP2/ERF43*, *AnAP2/ERF55*, *AnAP2/ERF84*, *AnAP2/ERF95*, *AnAP2/ERF127* and *AnAP2/ERF135* were downregulated (Figure 8). Under GA treatment, compared with CK, *AnAP2/ERF18*, *AnAP2/ERF34*, *AnAP2/ERF55* and *AnAP2/ERF127* were upregulated while the remaining eight genes were downregulated (Figure 8a,c,f,k). Under ABA treatment, *AnAP2/ERF18*, *AnAP2/ERF43*, *AnAP2/ERF55*, *AnAP2/ERF63* were downregulated, with all others upregulated (Figure 8a,d,f,g). These results indicate that *AnAP2/ERF* family members may have divergent biological functions in response to hormonal stimuli, and their expression profiles provide valuable insights for assessing their physiological roles.

### 3.7. Functional Diversification of AP2/ERF Transcription Factors: DNA-Binding Conservation and Stress-Adaptive Specialization

This study systematically characterized the biological properties of 12 *AnAP2/ERF* transcription factors through functional annotation: All genes harbored the conserved AP2 DNA-binding domain (Pfam: PF00847.19) and exhibited DNA-binding transcription factor activity (GO:0003700), with 75% (9/12) participating in the ethylene-activated signaling pathway (GO:0009873). Functional divergence was observed among key members—*AnAP2/ERF95* regulated thermotolerance (GO:0010286), dehydration response (GO:0009414), and oxidative stress processes; *AnAP2/ERF22* mediated insect defense (GO:0002213); while *AnAP2/ERF135* possessed dual features of membrane localization (GO:0016020) and glucosinolate metabolic function (GO:0019760) (Table S3). These findings demonstrate the pivotal roles of this gene family in stress adaptation and developmental regulation.

## 4. Discussion

*AP2/ERF* superfamily genes are important candidates for improving plant growth and tolerance to abiotic stresses such as drought, low temperature, and high temperature. Therefore, genome-wide studies of this gene family help elucidate its roles in plant development, molecular processes, and stress resistance mechanisms [4,39,40]. However, no genome-wide analysis of the *AP2/ERF* superfamily has been reported for *A. nelumboides*. Here, we present the first comprehensive study of the *AP2/ERF* superfamily in *A. nelumboides*, including phylogenetic analysis, gene structure characterization, motif distribution, chromosomal localization, and stress/hormone response profiling.

We identified 163 *AnAP2/ERF* genes, a number higher than in *A. thaliana* (147) and comparable to rice (162) [9,41]. The phylogenetic tree constructed with *A. thaliana AP2/ERF* proteins revealed evolutionary relationships and potential functional similarities of *AnAP2/ERF* genes (Figure 2), providing a reference for future functional studies. Motif and conserved domain analyses further support functional predictions (Figure 1). Given that tissue-specific expression correlates with gene function, we examined 12 selected *AnAP2/ERF* genes in *A. nelumboides* leaves, rhizomes, and roots via qRT-PCR. Seven genes (*AnAP2/ERF18*, *AnAP2/ERF43*, *AnAP2/ERF46*, *AnAP2/ERF55*, *AnAP2/ERF63*, *AnAP2/ERF84*, *AnAP2/ERF127*) showed root-specific high expression (Figure 6a,d–h,k), suggesting potential roles in root development and regulation.

*Cis*-regulatory elements (CREs) play key roles in regulating *AP2/ERF* gene expression, enabling the establishment of complex regulatory networks under stress conditions [19]. Promoter analysis revealed that *AnAP2/ERF* promoters contain hormone-responsive elements for IAA, GA, and ABA (Figure 4). Auxin biosynthesis and transport are linked to drought response, e.g., *YUC6/YUC7* overexpression enhances drought tolerance across species, as does exogenous auxin application, whereas *yuc1 yuc2 yuc6* mutants show increased drought sensitivity [42,43,44,45]. In rice, exogenous IAA boosts ROS scavenging capacity and upregulates auxin biosynthesis/transport genes without affecting catabolism genes [46]. Similarly, *Aux/IAA* genes modulate drought tolerance in alfalfa as positive/negative regulators [47]. When applied to *A. nelumboides*, exogenous IAA induced differential expression of 12 *AnAP2/ERF* genes (Figure 8). This aligns with *AP2/ERF’s* established role in environmental stress responses, exemplified by tobacco ntrav4 mutants upregulating ROS/proline/stress genes under osmotic stress [48]. Consistent with this, qRT-PCR confirmed differential expression of *AnAP2/ERF* genes under drought/flooding stress (Figure 7), suggesting IAA-mediated regulation of abiotic stress responses in *A. nelumboides*.

Studies indicate that GA levels and signaling are associated with abiotic stresses including drought, flooding, temperature extremes, and salinity [49]. In *A. thaliana*, overexpression of *EguGA20ox1/2* enhanced vegetative growth but increased stress sensitivity, whereas *EguGA2ox1*-OE plants exhibited strong stress tolerance [50]. Under drought conditions, *Haloxylon ammodendron* and *Haloxylon persicum* showed opposing trends in auxin and GA content, yet both species upregulated *AP2/ERF* transcription factors [51]. Similarly, *Pinus koraiensis* elevated GA levels and upregulated *AP2/ERF* expression under light stress [52]. Here, we observed that 12 *AnAP2/ERF* genes responded not only to drought/waterlogging stress but also showed differential expression under GA induction (Figure 7 and Figure 8), suggesting GA-mediated regulation of abiotic stress responses in *A. nelumboides*.

ABA plays pivotal roles in plant abiotic stress responses [53]. The ABA signaling pathway mediates stress adaptation, as evidenced by PeuPP2C members conferring salt/cold tolerance in *Populus euphratica* [54], *BnSnRK2* regulating drought response in *Brassica napus* [55], and *AtABF3* overexpression enhancing heat tolerance in *A. thaliana* [56]. Consistent with this, *RAP2.6* modulates abiotic stress via ABA-dependent pathways in *A. thaliana* [57], while soybean *GmERF113* overexpression elevates ABA levels and drought tolerance [58]. Beyond ABA responsiveness, the 12 *AnAP2/ERF* genes exhibited differential expression under drought/flooding stress (Figure 7 and Figure 8), suggesting ABA-mediated regulation of abiotic stress adaptation in *A. nelumboides*.

This study systematically revealed the pivotal role of the *AnAP2/ERF* transcription factor family in stress response and hormonal signaling through integrated functional annotation and expression profiling. Functional annotation demonstrated that all 12 genes harbor the conserved AP2 DNA-binding domain (Pfam: PF00847.19), with 75% (9/12) participating in the ethylene signaling pathway (GO:0009873). Key members exhibited functional divergence: *AnAP2/ERF95* was annotated to regulate dehydration response (GO:0009414), thermotolerance (GO:0010286), and oxidative stress processes [59], while *AnAP2/ERF135* was associated with glucosinolate metabolism (GO:0019760) (Table S3) [60]. These predictions were experimentally validated: under drought stress, AnAP2/ERF95 was significantly downregulated (Figure 7h), suggesting its role as a negative regulator in water-deficit response [61]. Conversely, under flooding stress and ABA treatment, *AnAP2/ERF95* was upregulated (Figure 7l) or suppressed (Figure 8f), respectively, indicating its response to osmotic stress via an ABA-independent pathway [62]. Notably, *AnAP2/ERF135* was upregulated under both drought and flooding stress (Figure 7l) while being suppressed by both IAA and ABA treatments (Figure 8), corroborating its dual functional annotation of membrane localization (GO:0016020) and defense metabolism (Table S3) [63]. This suggests its potential role in integrating environmental stress and hormonal signals to regulate secondary metabolism [64]. Furthermore, heterogeneous hormonal responses revealed functional specialization among these genes: *AnAP2/ERF55* (annotated to ethylene signaling) showed consistent downregulation under drought and ABA treatments (Figure 7f and Figure 8f) but upregulation under GA, indicating its role in balancing stress responses through ABA/GA signal antagonism (Table S3) [49]. In contrast, ERF22 (GO:0002213, insect defense) was upregulated under both flooding and IAA treatment (Figure 7b and Figure 8b), implicating its involvement in flood-induced defense mechanisms (Table S3) [65]. Although some discrepancies existed between qRT-PCR and transcriptome data (potentially due to sampling timepoints or biological replicates), multi-omics evidence collectively supports functional modularity within this gene family for environmental adaptation. While the core DNA-binding domain (GO:0003700) maintains conserved transcriptional activity (Table S3), divergent regulatory networks (e.g., *AnAP2/ERF95* multi-stress coordination and *AnAP2/ERF135* metabolic integration) provide the molecular basis for species-specific environmental adaptation (Table S3).

In summary, this study preliminarily confirmed that these *AnAP2/ERF* genes play an important role in the response to flooding and drought stress.

## 5. Conclusions

In this study, 163 *AnAP2/ERF* genes were identified in *A. nelumboides*, including 12 that simultaneously responded to drought and waterlogging stress with tissue-specific expression patterns. These 12 *AnAP2/ERF* genes also exhibited hormone responsiveness to IAA, GA, and ABA. This work provides novel insights into abiotic stress adaptation mechanisms in *A. nelumboides* and establishes a foundation for elucidating how *AnAP2/ERF* genes regulate stress tolerance in this species.

## Data Availability

The original contributions presented in this study are included in the article/Supplementary Material. Further inquiries can be directed to the corresponding author.

## Supplementary Materials

The following supporting information can be downloaded at: https://www.mdpi.com/article/10.3390/life15081269/s1, Figure S1: Evolution and gene structure analysis of *AnAP2/ERF* gene in *Adiantum nelumboides*; Table S1: 163 *AP2/ERF* family genes and related information of *A. nelumboides*; Table S2: Primer names and sequences involved in this study; Table S3: GO and KEGG analysis of *AnAP2/ERF* gene in *A. nelumboides*.
