# Supplementary figures and images for: Genome-Wide Identification and Comprehensive Analysis of AP2/ERF Gene Family in Adiantum nelumboides Under Abiotic Stress
Source: Life (Basel). 2025 Aug 11;15(8):1269. doi: 10.3390/life15081269 (PMC12387245; doi:10.3390/life15081269)

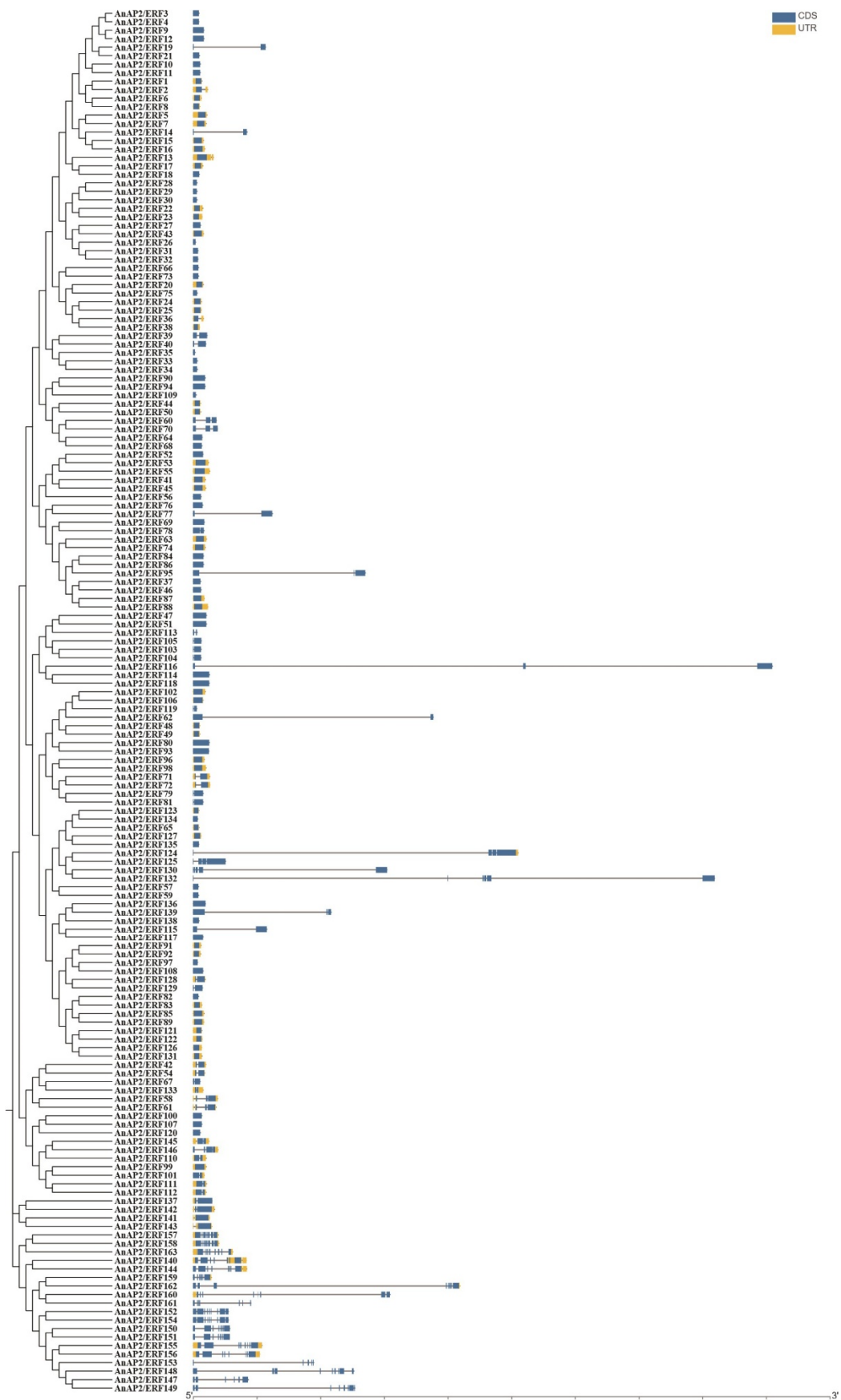

Figure. S1 Evolution and gene structure analysis of *AnAP2/ERF* gene in *Adiantum nelumboides*.

Supplement: Supplementary file 1 [file life-15-01269-s001.zip › Figure S1.pdf]
